# Bioactive Metabolites and Potential Mycotoxins Produced by *Cordyceps* Fungi: A Review of Safety

**DOI:** 10.3390/toxins12060410

**Published:** 2020-06-19

**Authors:** Bo Chen, Yanlei Sun, Feifei Luo, Chengshu Wang

**Affiliations:** 1 Key Laboratory of Insect Developmental and Evolutionary Biology, CAS Center for Excellence in Molecular Plant Sciences, Shanghai Institute of Plant Physiology and Ecology, Chinese Academy of Sciences, Shanghai 200032, China; chenbo@sippe.ac.cn (B.C.); sunyanlei@sippe.ac.cn (Y.S.); ffluo@sibs.ac.cn (F.L.); 2 CAS Center for Excellence in Biotic Interactions, University of Chinese Academy of Sciences, Beijing 100049, China; 3 School of Life Science and Technology, ShanghaiTech University, Shanghai 201210, China

**Keywords:** *Cordyceps* fungi, mass production, mycotoxins, biosynthetic gene cluster, toxicity, safety

## Abstract

Ascomycete *Cordyceps* fungi such as *C. militaris*, *C. cicadae*, and *C. guangdongensis* have been mass produced on artificial media either as food supplements or health additives while the byproducts of culture substrates are largely used as animal feed. The safety concerns associated with the daily consumption of *Cordyceps* fungi or related products are still being debated. On the one hand, the known compounds from these fungi such as adenosine analogs cordycepin and pentostatin have demonstrated different beneficial or pharmaceutical activities but also dose-dependent cytotoxicities, neurological toxicities and or toxicological effects in humans and animals. On the other hand, the possibility of mycotoxin production by *Cordyceps* fungi has not been completely ruled out. In contrast to a few metabolites identified, an array of biosynthetic gene clusters (BGCs) are encoded in each genome of these fungi with the potential to produce a plethora of as yet unknown secondary metabolites. Conservation analysis of BGCs suggests that mycotoxin analogs of PR-toxin and trichothecenes might be produced by *Cordyceps* fungi. Future elucidation of the compounds produced by these functionally unknown BGCs, and in-depth assessments of metabolite bioactivity and chemical safety, will not only facilitate the safe use of *Cordyceps* fungi as human food or alternative medicine, but will also benefit the use of mass production byproducts as animal feed. To corroborate the long record of use as a traditional medicine, future efforts will also benefit the exploration of *Cordyceps* fungi for pharmaceutical purposes.

## 1. Introduction

Filamentous fungi are rich producers of bioactive secondary metabolites, some of which either have been developed as commercial drugs to save lives or are carcinogenic or neurotoxic mycotoxins that threaten human health [1]. The production of these compounds has long been considered to be dispensable for fungal biology since the disruption of the biosynthetic gene clusters (BGCs) could barely, if at all, impair fungal growth and development under experimental conditions [2]. However, many studies have shown that the small molecules produced by fungi play essential roles in fungus–environment interactions [3,4]. For example, metabolites with antibiotic and antifungal activities are used by the producing fungi to outcompete other microbes [5,6]. The phytotoxins produced by plant pathogens are required in mediating fungus–plant interactions [7], and insecticidal toxins biosynthesized by insect pathogens facilitate fungal infection of insect hosts [8,9]. In addition, the mycotoxins produced by plant pathogens or endophytes can frequently result in toxic effects at different levels in herbivorous animals [10], which has been considered as a strategy employed by plants against grazers [11]. Thus, small molecules produced by fungi are of biological importance to producers and beyond.

In nature, there are more than 1000 species of fungi that can infect and kill insects, most of which are ascomycete entomopathogenic fungi [12]. In particular, three families of Hypocrealean fungi, i.e., Cordycipitaceae, Ophiocordycipitaceae, and Clavicipitaceae, contain species that have been used either for biocontrol of insect pests or as Traditional Chinese Medicine (TCM) in Asian countries and beyond [13,14]. For example, the species of *Ophiocordyceps sinensis*, *Cordyceps militaris*, and *C. cicadae* (syn. *Isaria cicadae*) have been used as TCM with recorded antibiotic, anti-inflammatory, anti-aging, anti-cancer, anti-proliferative, anti-metastatic, anti-fatigue, and immunomodulatory activities or effects (Table 1) for a long time in history [15,16]. On the other hand, similar to other traditional herbs, the safety of consuming these fungi has long been a concern [16]. A plethora of bioactive metabolites have been identified from Hypocrealean entomopathogens [14,17,18], including those which have been developed as commercial drugs such as the immunosuppressant drug cyclosporin A, a cyclodepsipeptide isolated from the Ophiocordycipitaceae fungus *Tolypocladium inflatum* [6] and the anti-leukemia drug pentostatin, first isolated from *Streptomyces*, found in *C. militaris* [19]. Apart from the medicinal or beneficial effects reported for the chemicals identified from *Cordyceps* fungi, side effects of cytotoxicity and or neurological toxicity have also been reported for these compounds (Table 1). Anecdotal records of nausea, diarrhea and even excessive post-extraction bleeding have been reported after daily consumption of *Cordyceps* fruiting bodies or related products [20,21]. Thus, in-depth safety evaluation is still required before consuming these fungi.

*Cordyceps* fungi are being mass produced for harvesting the fruiting bodies for food and health additives while the byproducts (largely culture substrates) of mass production are then mostly used as animal feed [22]. To alleviate the safety concerns for both purposes, this paper reviews the production and biological activity/toxicity of known metabolites identified from *Cordyceps* fungi and unknown metabolites deduced from the conserved biosynthetic gene clusters (BGCs) by comparative analysis with those BGCs involved in producing known mycotoxins based on the obtained genome information of these fungi [23,24,25]. The content of literature reviews conducted in the paper may benefit the future exploration and safety assessment of *Cordyceps* fungi and their related byproducts used for food, traditional medicine or animal feed.

## 2. Mass Production of *Cordyceps* Fungi

**Figure 1:**
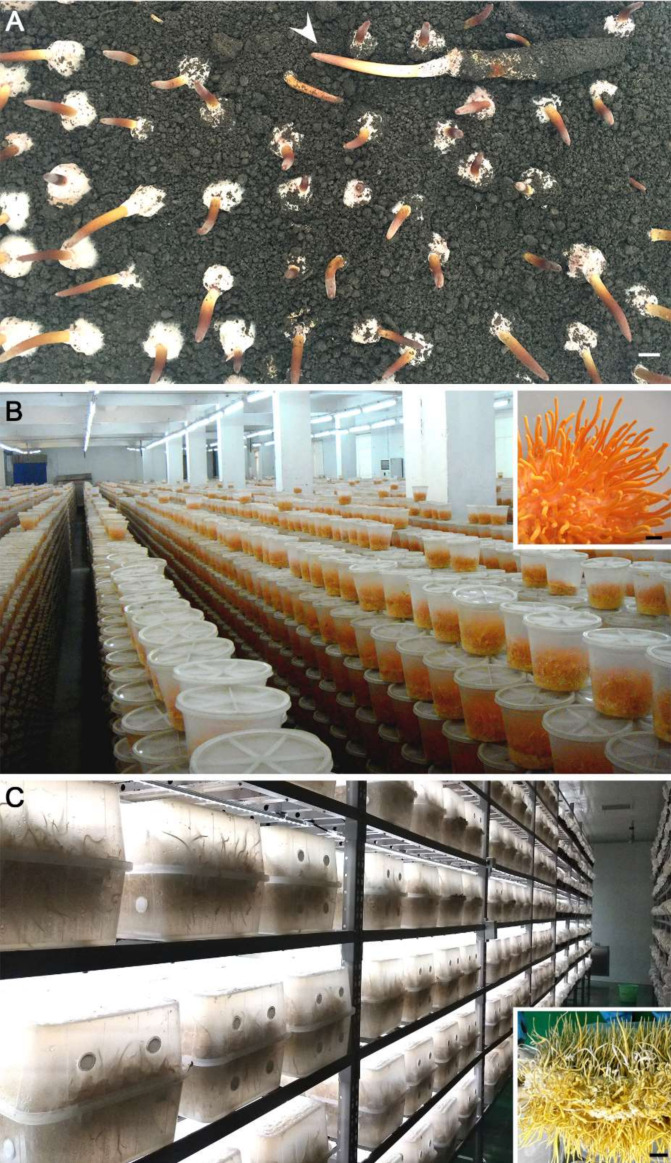
Mass production of *Cordyceps* fungi. (**A**) Successful induction of the fruiting bodies (arrowed) of *O. sinensis* after inoculation of the ghost moth larvae for more than 150 days (image taken from the Sunshine Lake LLC, Yichang, China). Bar, 1 cm. (**B**) Mass production of *C. militaris* in plastic bottles (image taken from the Honghao Biotech Company, Jiangmeng, China). Insert, the fruiting bodies formed in a bottle 45 days post-inoculation. Bar, 1 cm. (**C**) Mass production of *C. cicadae* in plastic boxes (image taken from the BioAisa Pharmaceuticals, Pinghu, China). Insert, the fruiting bodies formed in a box 20 days post-inoculation. Bar, 1 cm.

Ascomycete entomopathogenic fungi are facultative with saprophytic growth abilities. However, it is still technically challenging, often difficult, to induce fungal sexual fruiting bodies on artificial media or on insect hosts under laboratory conditions [26]. Until recently, induction of the fruiting-body formation of the caterpillar fungus *O. sinensis* (best known as *C. sinensis*, one of the most expensive traditional medicines) was not successful [27,28]. After inoculation of the ghost moth (*Hepialus* spp.) larvae, it is an energy-intensive process for fungal infection and development within the insect body cavity at a relatively low temperature (less than 18 °C) and the formation of fruiting bodies for a total period of more than half a year (Figure 1A). Dozens of tons of the fruiting bodies (attached with insect cadavers) can now be produced annually in China [28]. In contrast, mass production of *C. militaris* has long been successful by inoculation of fungal propagates on artificial media (e.g., rice medium) (Figure 1B). Different from the homothallic nature of *O. sinensis* [26], *C. militaris* is sexually heterothallic but its single mating-type can also fruit but without mating and meiosis to produce sexual perithecia [29]. Without considering the mass production of *C. militaris* in Japan, South Korea and Vietnam, the annual yield of the dried fruiting bodies reaches up to 10,000 tons per year in China [22]. The fruiting bodies of *C. cicadae* together with the mycosed cicada pupae have also been used as a TCM in renal protection or for the treatment of chronic kidney disease [23]. Mass production of this fungus has also been successful with a yield of hundreds of tons each year (Figure 1C). In contrast to *C. militaris*, asexual fruiting bodies, i.e., synnema-like structures, are largely produced by *C. cicadae* [23]. A few other species such as *C. guangdongensis* have also been mass produced at different magnitudes in Asian countries. Without consideration of the liquid fermentations of *Cordyceps* fungi [22], careful evaluation and safety assessment are still required regarding the (daily) consumption of enormous amounts of fruiting bodies and their related products as foods or health-promoting additives, and utilization of the leftover substrates as animal feed.

## 3. Known Metabolites Produced by *Cordyceps* Fungi

**Figure 2:**
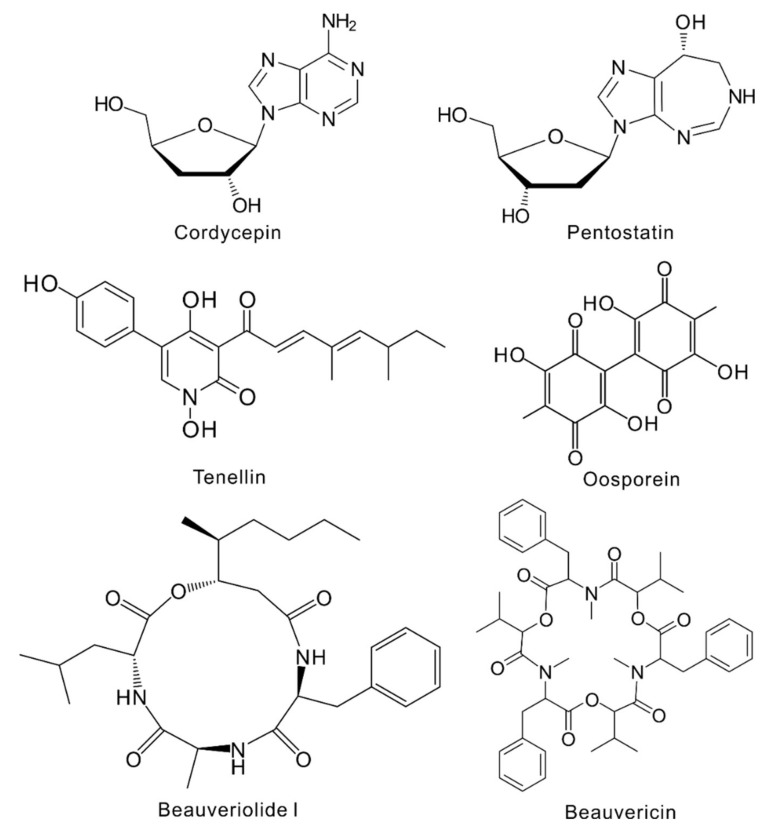
Structure of the selected metabolites identified from *Cordyceps* fungi.

Different compounds have been isolated from entomopathogenic fungi including *Cordyceps* species [17]. One of the most important but arguable metabolites produced by *C. militaris* is cordycepin, i.e., the adenosine analog 3’-deoxyadenosine (Figure 2). This compound is not produced in *O. sinensis* and *C. cicadae;* elucidation of its biosynthetic mechanism showed that the responsible BGC is only present in *C. militaris* and *C. kyusyuensis* as well as the evolutionarily distant mold fungus *Aspergillus nidulans* [19]. It was later found that the field-collected samples of *O. sinensis*, i.e., the complex of fruiting body and insect cadaver, were frequently contaminated with *A. nidulans* and even *C. militaris* that might contribute to the detection of trace amounts of cordycepin in caterpillar fungus [30]. Cordycepin can inhibit RNA synthesis and has demonstrated immense medicinal potential (Table 1), including anti-cancer, anti-inflammatory, antibiotic, anti-virus, and antioxidant activities [31]. However, in addition to its dosage-dependent toxicity to different cells, cordycepin has shown an effect in stimulating testosterone production in the models of both mouse Ledydig cells and mice, which may alter male fertility [32]. Recently, it has been shown that the anti-leukemia drug pentostatin (Figure 2), i.e., the 2’-deoxycoformycin originally isolated from *Streptomyces antibioticus* being an irreversible inhibitor of adenosine deaminase, can also be produced by *C. militaris* through the same BGC for cordycepin production via a protector–protégé strategy [19]. Similar to other chemotherapeutic drugs/agents, dosage- and schedule-dependent side effects have also been observed for pentostatin that include nausea, diarrhea, and renal and neurological toxicities [33]. The test of cordycepin in combination with pentostatin can trigger severe gastrointestinal toxicity and bone marrow toxicity in dogs [34].

Apart from cordycepin and pentostatin, another adenosine analog *N*^6^-(2-Hydroxyethyl)-adenosine (HEA) has been identified in *C. cicadae*, *C. militaris*, and other species of *Cordyceps* with renal protection and anti-cancer activities [35,36]. Insecticidal activity of HEA has also been demonstrated by targeting the adenosine receptor (AdoR) of insects [37], suggesting that adenosine analogs can be recognized by AdoR(s) [38]. A different family of AdoRs, the G-protein coupled receptors with seven transmembrane domains, has been identified in humans as potential drug targets [39]. Since adenosine is multifunctional in the physiology of different organisms, the functions of cordycepin and HEA are still unclear, notably whether they act as agonists or antagonists of AdoRs in mammals. Deletion of *AdoRa1* in mice has led to decreased fertility and an increased risk of seizures [38]. The long-term effect of *Cordyceps* consumption and the effects of cordycepin and HEA administration on activation or inactivation of AdoRs require further investigation.

Other known metabolites produced by either *C. militaris* or *C. cicadae* include 2-pyridone alkaloid tenellin-like compounds and the bibenzoquinone oosporein (Figure 2). Different structures of Tenellin-like pyridones have been identified in different Cordycipitaceae fungi including fumosorinone produced by *C.* (*Isaria*) *fumosorosea* [40], farinosones by *I. farinosus* [41], and militarinones by *C. militaris* [42]. These 2-pyridones (Table 1) can maintain iron hemostasis and have also shown profound neuritogenic activity and cell cytotoxicities [42]. Oosporein, originally identified from the insect pathogen *Beauveria bassiana* (Syn. *Cordyceps bassiana*), shows insecticidal and antibiotic activities that promote fungal infection of insect hosts [9,43]. The conserved gene cluster and production of oosporein has been detected in *C. cicadae* [23]. This compound can also cause gout in avian species including chickens, turkeys and other birds, and can therefore threaten the safety of the poultry industry if contaminated substrates are used as feed [44].

A few cyclodepsipeptides (Figure 2) have also been identified from *Cordyceps* fungi, e.g., beauveriolide I and III from *C. militaris* [45] and beauvericin from *C. cicadae* [23,46]. Beauveriolides have demonstrated anti-aging [47], beta-amyloid-lowering [48], and anti-atherogenic activity by inhibition of lipid droplet accumulation in macrophages without any obvious side effects [49]. However, insecticidal and nematicidal beauvericin, first isolated from *B. bassiana*, can induce cytotoxicity and cell apoptosis in a dose-dependent manner due to its ionophoric property that can increase ion permeability in membranes [50]. Overall, along with the beneficial medicinal and or biological activities, different negative effects are also evident with the compounds identified from *Cordyceps* fungi that raise safety concerns about its consumption.

## 4. Unknown Mycotoxins May be Produced by *Cordyceps* Fungi

**Figure 3:**
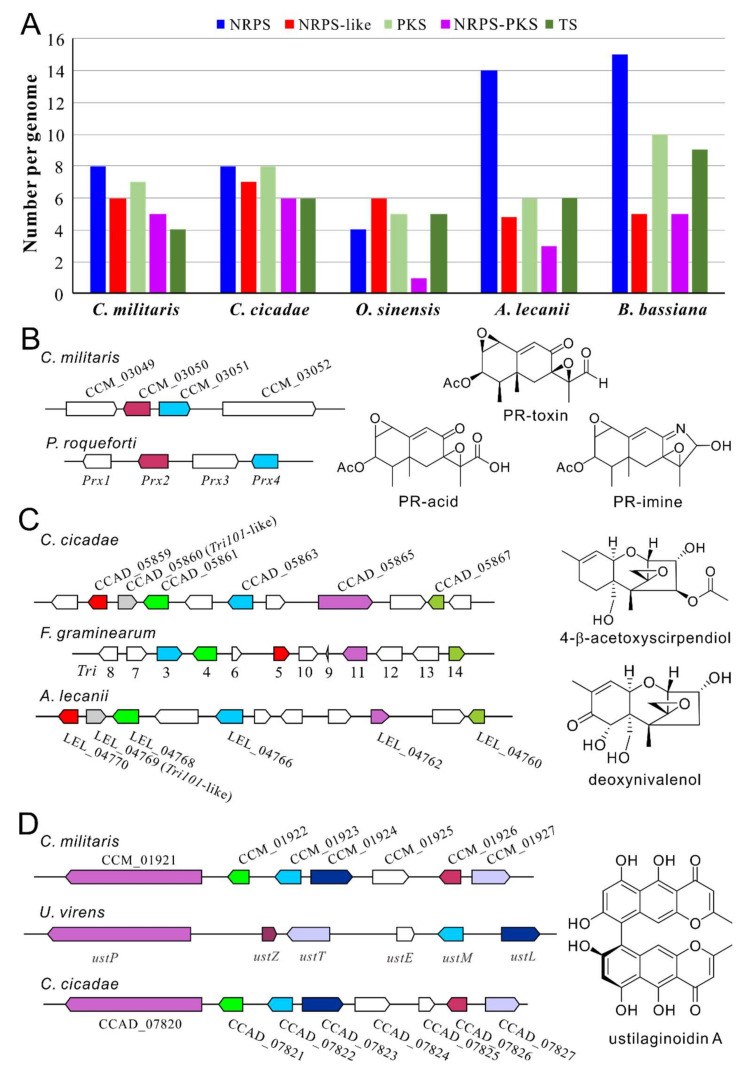
Conservation analysis of the gene clusters between *Cordyceps* and other fungi involved in toxin production. (**A**) Comparative analysis of the biosynthetic gene clusters (BGCs) encoded in the genomes of the selected Cordycipitaceae fungi. NRPS, non-ribosomal peptide synthetase; PKS, polyketide synthase; TS, terpene synthase. (**B**) Conservation between the gene clusters of *C. militaris* and those of *P. roqueforti* involved in PR-toxin production. (**C**) Conservation between the gene clusters of the Cordycipitaceae fungi *C. cicadae* and *A. lecanii* and that of *F. graminearum* involved in the biosynthesis of trichothecenes. (**D**) Conservation between the gene clusters of *C. militaris* and *C. cicadae* and that of *U. virens* involved in the production of ustilaginoidins. The genes labeled in the same color within the panels (**B**–**D**) represent orthologous relationships. The compound(s) shown in the right of the panels (**B**–**D**) indicates the structures of the representative metabolites produced by the known BGCs in the related or reference fungal species.

Apart from the metabolites described above, many more can be expected after genomic analysis of *Cordyceps* fungi that identifies an array of BGCs encoded in each fungus (Figure 3A). For example, eight non-ribosomal peptide synthetase (NRPS), seven polyketide synthase (PKS), five NRPS-PKS and four terpene synthase (TS) BGCs are encoded in the genome of *C. militaris* [25,51]. Likewise, the BGCs of eight NRPSs, eight PKSs, six NRPS-PKSs and six TSs are encoded in *C. cicadae* [23]. Consistently, metabolomic analysis also suggested that diverse compounds can be produced by these fungi [23,52]. Due to the frequent occurrence of gene silence of fungal BGCs under experimental conditions [2], many unknown metabolites remain to be determined. Genome-wide phylogenetic, gene cluster content, and core enzyme structure analyses of NRPS, PKS, and NRPS-PKS BGCs suggest that the *Cordyceps* fungi may not produce carcinogenic mycotoxins such as aflatoxins [23,25]. However, our further analysis indicated that the putative aristolochen synthase CCM_03050 is highly similar (65% identity at amino acid level) to PRX2, and the clustered CCM_03051 is similar to the short chain dehydrogenase (SDR) PRX4 of *Penicillium roqueforti* involved in PR-toxin production (Figure 3B). The bicyclic sesquiterpene PR-toxin can cause significant damage to liver and kidney, induce abortions and reduce fertility in cattle. However, its derivatives such as PR-acid and PR-imine are largely non-toxic and unstable [53]. The biosynthesis of PR-toxin requires at least four genes, i.e., *Prx1-Prx4* in *P. roqueforti* [54]. Considering that the tailoring enzyme genes *Prx1* (for a SDR) and *Prx3* (for a quinone oxidoreductase) are missing in *C. militaris* whereas the clustered *CCM_03049* encodes a putative dioxygenase/oxygenase and *CCM_03052* encodes a cytochrome P450 enzyme, this TS BGC may not biosynthesize PR-toxin but its analog(s) requiring further investigation. Intriguingly, these conserved genes are absent in the genomes of the closely-related *C. cicadae* and other Cordycipitaceae fungi, based on the survey of their genome contents.

We also found that the Cordycipitaceae fungi *C. cicadae* and *Akanthomyces lecanii* (anamorph: *Lecanicillium lecanii*) each encode a gene cluster that shows conservation with the BGC involved in the production of trichothecene (Tri) mycotoxins in wheat head blight *Fusarium graminearum* (Figure 3C). Phytotoxic trichothecenes are a class of sesquiterpenes that contain more than 150 chemically-related compounds such as type A mycotoxins T-2, HT-2, and NX-2, and type B deoxynivalenol (DON), acetylated DON, and nivalenol toxins that can inhibit protein synthesis and are neurotoxic, immunosuppressive, and nephrotoxic in mammals [55,56]. The biosynthesis of trichothecenes has been clarified with the involvement of 15 *Tri* genes located at three different loci including a 12-gene core cluster, a single gene *Tri101* and two genes *Tri1* and *Tri16* loci on different chromosomes of *F. graminearum* [57,58]. We found that the homologs of the core gene *Tri5* for trichodiene synthase are conservedly present in *C. cicadae* (CCAD_05859, 70% identity) and *A. lecanii* (LEL_0770, 79% identity). The homologs of the essential biosynthetic genes *Tri3*, *Tri4*, *Tri11* and *Tri14* are also present in the genomes of these two fungi. In addition, interestingly, the homologs of the isolated gene *Tri101* (encoding an acetyltransferase) in *F. graminearum* are present in the core cluster of *Cordyceps* fungi, i.e., *LEL_04769* (40% identity) and *CCAD_05860* (37% identity) (Figure 3C). Likewise, the homologs of the isolated gene *Tri1* are also present in the separated loci of two fungi (*CCAD_02575*, 42% identity; *LEL_06734*, 41% identity). Taken together, it is considerably likely that *C. cicadae* and *A. lecanii* may produce trichothecene-like mycotoxin(s) that requires clarification and toxicity tests. In support, the trichothecene derivative 4-β-acetoxyscirpendiol (4-acetyl-12,13-epoxyl-9-trichothecene-3,15-diol) was once isolated from the fruit-body samples of *Isaria japonica* (syn. *Cordyceps tenuipes*) and the compound could induce apoptosis of human leukemia cells [59]. This *Tri*-like BGC is not present in *C. militaris* but is present in the closely-related biocontrol fungus *B. bassiana* (TRI5 vs. BBA_08696, 69% identity; TRI101 vs. BBA_08697, 41%) after genome survey [56]. A *Tri*-like BGC has also been identified in diverse fungal species including *Trichoderma* spp., which is responsible for the production of the trichothecene derivatives harzianums A and B with antimicrobial activities [60,61].

The bis-naphthopyrone-type pigments are widely produced by different fungi that can protect filamentous fungi from fungivores [62] or abiotic stress factors like UV radiation and high temperatures [63,64]. Genome survey indicated that both *C. militaris* and *C. cicadae* contain a conserved PKS BGC like that of rice false smut *Ustilaginoidea virens* (Figure 3D), which is responsible for the production of ustilaginoidins [65]. Different analogs of ustilaginoidins have varied level of antibacterial, cytotoxic, and phytotoxic activities [66]. The exact compound(s) produced by this PKS gene cluster remains to be determined in *Cordyceps* fungi.

In contrast to other *Cordyceps* fungi, the caterpillar fungus *O. sinensis* has a highly repetitive genome with a limited number of genes and BGCs (Figure 3A) [24]. Besides the reported bioactive constituents like nucleosides, sterols and polysaccharides [18,67,68,69], a few compounds have been identified from this fungus with different activities such as the aurantiamides cordyceamides A and B with unclear activity/toxicity [70], the cyclodipeptide cordycedipeptide A with cytotoxicity [71], and the cyclodipeptide cordysinins with antioxidant activity [69,72]. Overall, the reputed health benefits of this fungus are still unclear [73]. Despite the mass production of sexual fruiting bodies being recently successful [74], the slow growing nature of this fungus makes it technically problematic to perform transgenic and chemical genetic investigations [73]. With the help of heterologous expression systems, future efforts can be taken to explore the production, if any, of mycotoxin as well as the pharmaceutical potential of this fungus.

## 5. Requirement of Safety Assessments

As indicated above, toxicological concerns about consuming *Cordyceps* fungi as food supplements or using their byproducts as animal feed are raised based on the evaluations of either the known compounds they produce or those previously unreported toxins being putatively produced by the conserved BGCs of *Cordyceps* fungi. Obviously, the production of the avian-gout toxin oosporein by *C. cicadae* suggests that mass-production byproducts cannot be used as feed for poultry. In particular, except for the unknown metabolites biosynthesized by other BGCs, the potential of the production of analogous PR-toxin in *C. militaris* and Tri-like toxin(s) in *C. cicadae* raises substantial concerns that require further investigation and safety assessments. Traditionally, instead of using the purified compounds, the fruiting bodies or mycelium samples of *Cordyceps* fungi were used for direct safety assessments, including bacterial Ames tests, different cell- and/or animal-model tests for mutagenic, clastogenic, genotoxic, and (sub-)accurate toxic effects, which suggested that consumption of *Cordyceps* fungi might be safe [75,76]. However, considering the magnitude of the mass production of *Cordyceps* fungi in China and other countries, in-depth monitoring and assessment are still required, given the daily consumption of *Cordyceps* fruiting bodies or related products as tonics or food/health additives and the use of culture substrates as feed. In particular, aside from the feature of culture stability, high titer of cordycepin and pentostatin production is being used as the key standard for screening of *C. militaris* strains for industrial mass production. Consistent with the side effects of pentostatin and adenosine analogs [33,38], the negative effects of nausea and diarrhea have been anecdotally reported by enthusiasts after consuming products with enriched contents of cordycepin/pentostatin [14]. Indeed, it is common and typical that mycotoxin production and accumulation are dependent on the fungal culturing media and stage, and the side effects of daily consumptions are dose- and even consumer-dependent [20]. For example, trichothecene production was not detected in *A. lecanii* and *B. bassiana* after inductions in multiple artificial media used for the successful induction of toxin formations in other fungi [56]. It is critical at least that toxin production is clarified for the *Cordyceps* fungi under mass production conditions.

## 6. Conclusions and Prospective

Along with the increasing level of the mass production of *Cordyceps* fungi such as *C. militaris* and *C. cicadae*, there is increasing consumption of the fruiting bodies or related products as food supplements or health additives, and use of the byproducts as animal feed. To alleviate safety concerns, full elucidation of the BGCs’ capacities in production of different compounds is critically needed. In addition to promoting toxicological tests with the known and newly-identified compounds, in-depth investigations may also benefit the exploration of these fungi for pharmaceutical potential.

## Figures and Tables

**Table 1:** Summary of the reported bioactivity and toxicity of the compounds identified from *Cordyceps* fungi.

Compound	Producing Fungus	Bioactivities	Toxic Effect
Cordycepin	*C. militaris*; *C. kyusyuensis*	Anticancer, anti- inflammatory, antioxidant, inhibition of RNA synthesis, insecticidal, antibiotic, antifungal, antivirus	Gastrointestinal toxicity, bone marrow toxicity, decrease in toxicity
Pentostatin	*C. militaris*	Immunosuppressive, inhibitor of adenosine deaminase, antineoplastic	Nausea, diarrhea, renal and neurological toxicities, pulmonary toxicity, gastrointestinal toxicity
N6-(2-Hydroxy- ethyl)-adenosine	*C. militaris*; *C. cicadae*	Renal protection, anti-cancer, insecticidal	Induction of oxidative stress
Tenellin	*C. bassiana*	Iron chelation, inhibitor of membrane ATPase	Toxic towards erythrocytes
Militarinones	*C. militaris*	Antimicrobial	Cytotoxicity
Fumosorinone	*C. fumosorosea*	Inhibitor of tyrosine phosphatase 1B, activation of insulin pathway, anti-diabetic	/
Farinosones	*C. farinosa*	Neuritotrophic activity	Cytotoxicity
Oosporein	*C. cicadae*; *C. bassiana*	Immunosuppressive, antimicrobial, metal detoxification	Cytotoxicity
Beauveriolides	*C. militaris*; *C. bassiana*	Anti-aging, beta-amyloid lowering, anti-atherogenic	Cytotoxicity
Beauvericin	*C. cicadae*; *C. bassiana*	Insecticidal, nematicidal, induction of cell apoptosis, ionophoric property	Cytotoxicity
Cordyceamides	*O. sinensis*	/	Cytotoxicity
Cordycedipeptide	*O. sinensis*	/	Cytotoxicity
Cordysinins	*O. sinensis*	Anti-inflammatory	/
